# Characteristics of oral microbiota in plateau and plain youth‐positive correlations between blood lipid level, metabolism and specific microflora in the plateau group

**DOI:** 10.3389/fcimb.2022.952579

**Published:** 2022-08-10

**Authors:** LiBo Zhao, Huanhuan Wang, Yinghui Gao, Benchuan Hao, Xueyan Li, Ruoqing Wen, Kaibing Chen, Li Fan, Lin Liu

**Affiliations:** ^1^ Cardiology Department of the Second Medical Center and National Clinical Research Center for Geriatric Diseases, Chinese People's Liberation Army General Hospital, Beijing, China; ^2^ College of Nursing, Peking University, Beijing, China; ^3^ Sleep Center, Peking University International Hospital, Beijing, China; ^4^ College of Basic Medicine, Gansu University of Traditional Chinese Medicine, Lanzhou, China; ^5^ College of Integrated Traditional Chinese and Western Medicine, Gansu University of Traditional Chinese Medicine, Lanzhou, China; ^6^ Sleep Center, The Affiliated Hospital of Gansu University of Traditional Chinese Medicine, Lanzhou, China; ^7^ Department of Pulmonary and Critical Care Medicine of the Second Medical Center and National Clinical Research Center for Geriatric Diseases, Chinese People's Liberation Army General Hospital, Beijing, China

**Keywords:** oral microbiota, high-throughput sequencing, biodiversity, altitude, relative abundance, metabolic function, triglyceride

## Abstract

**Objectives:**

To analyze the characteristics of oral microbiota in plateau and plain youth and the possible function of the microbiome.

**Materials and methods:**

A total of 120 healthy young males (80 on the plateau, 40 on the plain) completed this cross-sectional study. Oral microflora samples were collected from all participants. The bacterial 16S rDNA was amplified using PCR and sequenced using Illumina MiSeq high-throughput sequencing. The data were analyzed to determine the microbial distribution and community structure of the oral microflora from the two groups. Metastats was used to test differences in relative species abundance between the groups. The correlation between the abundance of specific bacteria and blood indicators was also analyzed.

**Results:**

As demonstrated by alpha and beta diversity, the plateau group had lower microbial richness and a less even distribution of oral microbiota than the plain group. All predominant phyla and genera were qualitatively similar between the two groups, but their relative abundances differed. The relative abundance of bacteria in the phylum Firmicutes was significantly higher in the plateau group than in the plain group. At the genus level, *Streptococcus* spp. and *Gemella* spp. were also more abundant in the plateau group. The functional prediction indicated vigorous microbial metabolism in the oral bacterial community. We also found that the relative abundance of *Streptococcus* spp., the dominant genus, was positively correlated with triglyceride levels in the plateau group.

**Conclusions:**

With increasing altitude, the diversity of oral microbiota and the relative proportion of predominant bacteria were altered. The distribution and related function of *Streptococcus* spp. were prominent in plateau samples. This comprehensive study of the relationship between oral microecology and elevation provides a point of reference for studying the human body’s adaptability or inadaptability to high altitude.

## Introduction

The body has at least 10 times as many microbes as our own cells (Lu et al., 2014). The human microbiome could be called the second human genome, and its composition is closely related to human disease and health. Microbiome imbalance, destroying the stability of the symbiotic microbiota composition, has a direct impact on human health, involving the host’s immune response and metabolism (Duran-Pinedo et al., 2021). The abundance of the human oral microbiome plays an important role in maintaining the balance of the oral environment and preventing tooth decay and other oral diseases, which is second only to that of the gastrointestinal tract. In addition, the change of oral microflora is associated with some diseases such as diabetes, bacteremia, endocarditis and cancer. Owing to the wide impact of oral microflora on the body and the convenience of sampling, oral microbiome was used for comparative study among different groups.

Many people live or travel to high altitudes and are exposed to low barometric pressure and low oxygen, which leads to a number of physiological responses, including changes in the microbial community (Dong et al., 2021). In some cases, however, adverse factors such as hypoxia can cause maladaptive reactions, lead to various forms of acute and chronic altitude disease, such as hypertension, heart failure, asthma, high altitude pulmonary edema, and even cognitive impairment (Mallet et al., 2021; Wuyam et al., 2022). The etiological study and ecological prevention of altitude sickness have gradually become the significant health issues. With the continuous development of molecular biology techniques, high-throughput sequencing has become an effective method for oral microflora analysis, which can better research the relationship between oral microbiota and high altitude.

Therefore, in this study, Illumina MiSeq high-throughput sequencing was used to compare the diversity and structure of oral microbial community between the healthy young people living on the plateau for one year and those on the plain. The study provides basic reference for further understanding of the microbial changes in altitude adaptation and novel methods of altitude diseases prevention and treatment (Harkins et al., 2020).

## Materials and methods

### Subjects selection

Eighty healthy males living for 12 months on a plateau (about 4000 meters above sea level, oxygen content 14.55%, atmospheric pressure 61.7KPa), who came from different places in China, were randomly selected as the group 1. In addition, randomly selected forty healthy men who were together for 12 months because of work constituted the group 2 in an area of North China (altitude 45 meters, oxygen content 20.87%, atmospheric pressure 100.8KPa). The inclusion criteria: (a) male between the ages of 18-25, (b) self-reported no organic disease, (c) good oral hygiene without bad eating habits. The exclusion criteria: (i) those who were taking lipid-lowering drugs, (ii) antibiotics had been used for 5 days or more within last 2 months, (iii) suffering from infectious diseases, contagion, endocrine systemic disease, or abnormal immune function, (iv) patients with benign and malignant tumors, (v) suffering from various oral diseases such as mouth abscesses, (vi) long-term heavy smokers. These participants were fully aware of the purpose of the study and signed informed consent. This study was reviewed by the Ethics Committee of PLA General Hospital (S2020-363-01).

### Acquisition of oral microflora samples and blood indexes

Routine blood measurements and biochemical analyses were conducted on samples collected from all study subjects (with their informed consent) during a physical examination. Measurements included red blood cell counts, blood lipid levels, total cholesterol (TC), triacylglycerol (TG), and aspartate aminotransferase (AST) levels. Blood samples and oral microflora samples were collected from study participants when they first awoke in the morning before they ate, drank, or brushed their teeth. A sterile cotton swab was thoroughly rubbed and rotated on the buccal mucosa, on the root of the tongue, and in the saliva. The tip of the swab was then placed in a cryopreservation tube and stored at -80°C.

### DNA extraction and PCR amplification

Total genomic DNA samples were extracted using the OMEGA Soil DNA Kit (M5635-02) (Omega Bio-Tek, Norcross, GA, USA), and stored at -20°C prior to further analysis. The quantity and quality of extracted DNA were measured using a NanoDrop NC2000 spectrophotometer (Thermo Fisher Scientific, Waltham, MA, USA) and 1.2% agarose gel electrophoresis, respectively. Forward primer 338F (5’-ACTCCTACGGGAGgCAGCA-3’) and reverse primer 806R (5’-GGACTACHVGGGTWTCTAAT-3’) were used for PCR amplification of the V3-V4 region of the bacterial 16S rRNA gene. Sample-specific barcode sequences were assigned. PCR components included 5μL buffer (5×), 0.25μL Fast PFU DNA polymerase (5U/μL), 2μL (2.5mM) dNTPs, 1μL (10uM) of each forward and reverse primers, 1μL DNA template and 14.75μL double-distilled H_2_O. The thermal cycle consisted of initial denaturation at 98°C for 5 min, followed by 24 cycles of denaturation at 98°C for 30 sec, annealing at 52°C for 30 sec, and extension at 72°C for 45 sec, with a final extension of 5 min at 72°C. PCR amplicons were purified with Vazyme V AHTSTM DNA Clean Beads (Vazyme, Nanjing, China) and quantified using the Quant-iT PicoGreen dsDNA Assay Kit (Invitrogen, Carlsbad, CA, USA).

### Illumina MiSeq sequencing

Amplicons were pooled in equal amounts, and the sequencing was performed using the Illlumina MiSeq platform (Illumina, San Diego, USA) with MiSeq Reagent Kit V3 at Beijing Qinglian Biotech Co.,Ltd. To ensure the sequencing quality, the optimal sequencing length of the target fragment was 200-450 bp. Before sequencing, Agilent High-Sensitivity DNA Kit was used to conduct quality inspection on Agilent Bioanalyzer. Make sure the qualified libraries had only one peak.

### Data processing and analysis

FLASH (https://sourceforge.net/projects/flashpage/Version 1.2.11) (Magoč and Salzberg, 2011) was used to splice the reads from each sample to obtain the original data. Then Mothur (https://mothur.org/Version 1.35.1) (Schloss et al., 2009) was used to remove sequences that were too short (≤200bp) or too long (≥500bp). Chimera was removed using UCHIME (Edgar et al., 2011) with GOLD dataset (Haas et al., 2011) as a reference. A high-quality sequence was then obtained.

Operational taxonomic units (OTUs) were clustered with a 97% similarity cutoff by USEARCH (http://www.drive5.com/usearch/Version 7.0) (Edgar, 2013). The RDP classifier (Wang et al., 2007) was used to systematically classify OTU sequences based on Bergey’s taxonomy, with the SILVA database (Pruesse et al., 2007) as a reference. Taxonomic classification can be divided into six levels above the species level: kingdom, phylum, class, order, family, and genus. The default threshold of annotated species analysis was 80% (the frequency of an OTU in the entire sample), below which a species was defined as unclassified. Alpha and beta diversity analyses were conducted on homogenized sample data based on OTUs. Phylogenetic investigation of communities by reconstruction of unobserved states (PICRUSt, version 1.0.0) (Langille et al., 2013) analysis showed that the gene function spectrum of corresponding bacteria could be predicted by the 16S rRNA gene sequence. Samples from the two groups were compared using Student’s t-test or the Mann-Whitney U test, and the proportions of counting data between groups were compared using a Chi-square test. Metastats (http://www.drive5.com/usearch/) (White et al., 2009) was used to compare differences in the relative abundance of species between plain and plateau groups. Spearman correlation analysis was used to examine the relationship between species abundance and blood-related indices. SPSS 25.0 software (SPSS Inc, Chicago, IL, USA) was used for all statistical analyses, with statistical significance between groups defined as *P* < 0.05.

## Results

### General characteristics

Both study groups consisted of young Chinese males. There were no statistically significant differences in age, education, or teeth-brushing habits between group 1 (the plateau group) and group 2 (the plain group). Most of the participants in both groups had high-school educations and brushed their teeth twice daily (**Table 1**).

**Table 1:** Comparison of general characteristics and Alpha diversity between the two groups.

	plateau group	plain group	t or χ^2^	*P* value
Age,year	19.6 ± 1.5	19.9 ± 1.0	-1.33	0.185
Ethnic Han,n(%)	73(91.3)	39(97.5)	0.820	0.365
Completion of high school,n(%)	69(86.3)	35(87.5)	0.036	0.849
Brush teeth twice a day,n(%)	69(86.3)	34(85.0)	0.276	0.871
nseqs	63657.8 ± 11781.0	57057.4 ± 11720.9	2.89	0.004
sobs	568.2 ± 138.4	619.1 ± 149.7	-1.85	0.067
Chao index	799.3 ± 178.4	828.4 ± 173.2	-0.85	0.397
Ace index	885.6 ± 197.7	890.9 ± 167.7	-0.15	0.884
coverage	99.7 ± 0.1	99.6 ± 0.1	1.79	0.075
Shannon index	1.9 ± 0.7	2.7 ± 0.9	-5.06	<0.001
Npshannon index	1.9 ± 0.7	2.8 ± 0.9	-5.05	<0.001
Simpson index	0.4 ± 0.2	0.2 ± 0.2	5.31	<0.001

nseqs, the number of sequences clustered to OTUs; sobs, the number of observed species.

### Species annotation of sequencing results

A total of 120 oral mucosal swab samples were collected from 80 plateau males and 40 plain males. Through high-throughput sequencing, the sequence was clustered into an OTU with a similarity greater than 97%. The average sequence number of oral microflora from all subjects was 83978, and the mean sequence length was 466 bp. Then the OTUs number of cluster was 4303, in which 38 phyla, 79 classes, 138 orders, 238 families and 406 genera were annotated.

### The alpha diversity analyses between the plateau and plain groups

The alpha diversity analyses between the plateau group and the plain group are outlined in **Table 1** and **Supplementary Figure 1**. Compared to the bacterial biodiversity in plain group, that in plateau group was observed to exhibit a significantly lower level, which was reflected in a lower diversity indexes such as shannon index (1.9 ± 0.7 vs 2.7 ± 0.9, *P* < 0.001), and npshannon index (1.9 ± 0.7 vs 2.8 ± 0.9, *P* < 0.001). On the contrary, the simpson index of the plateau group was higher than that of the plain group (0.4 ± 0.2 vs 0.2 ± 0.2, *P* < 0.001).

**Figure 1:**
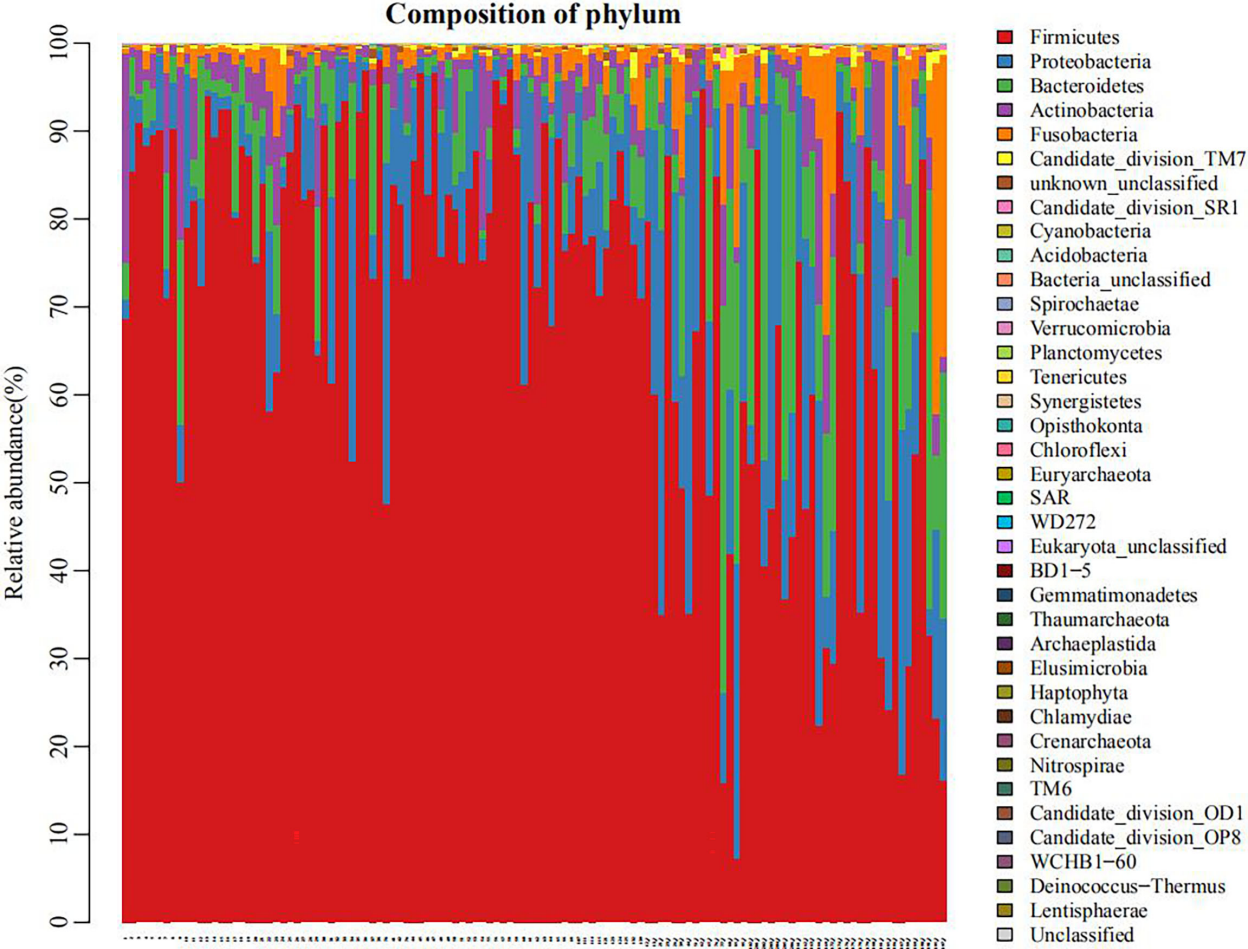
Histogram of flora distribution (taking phyla for example) The points on the horizontal coordinate represented individual objects of study. Different colors indicated different phyla, and the length of each colored band suggested the amount of specific phylum in an individual.

As shown in **Figure 1**, the microbial species in the plain group (the right third of the graph) were more evenly distributed than in the plateau group. In **Supplementary Figure 2**, the rank abundance curve reflects the richness and evenness of samples. The larger the value on the horizontal axis, the higher the OTU richness. The flatter the curve, the more even the OTU distribution. These results show that the distribution of microbial species in the plain group (group 2) was more uniform than that in the plateau group (group 1).

### The beta diversity analyses

**Figure 2:**
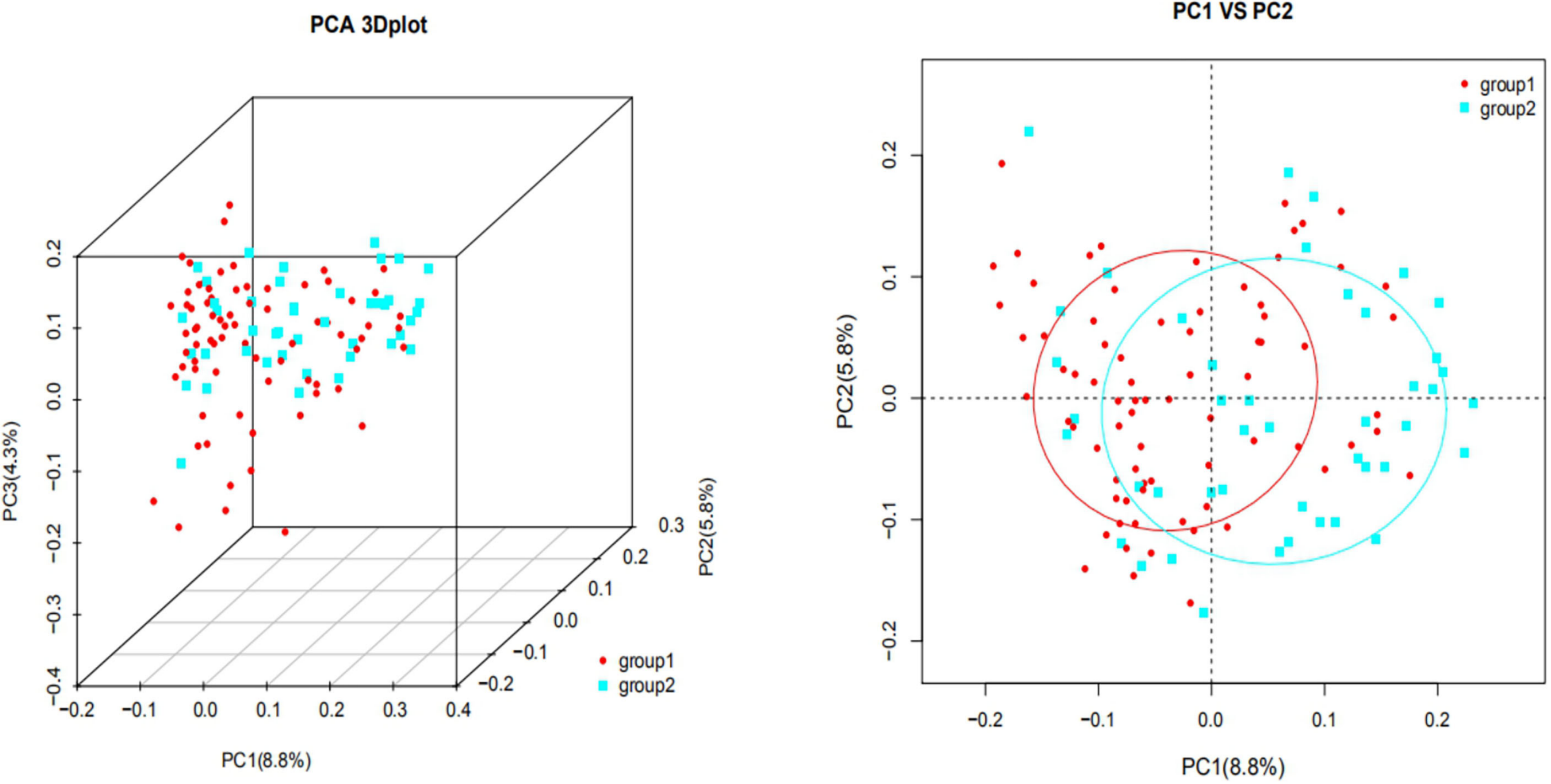
UniFrac ranking diagram based on principal coordinate analysis [2A (Left): three-dimensional, 2B (Right): two-dimensional. group 1: plateau group; group 2: plain group) The similarity and dissimilarity of bacterial community structures between the two groups were evaluated by PCoA that based on Bray-Curtis distances at the OTU level at 97% identity. The distribution of the two groups overlapped partially. PC1 explained 8.8% of the variation observed, and PC2 explained 5.8% of the variation.

**Figure 2** shows that each sample was represented by a dot, with circles of different colors representing different groups. The similarity and dissimilarity of bacterial community structures between the two groups were evaluated by PCoA that based on Bray-Curtis distances at the OTU level at 97% identity. The distribution of the two groups overlapped partially, but there were also obvious separations. PC1 explained 8.8% of the variation observed. PC2 and PC3 explained 5.8% and 4.3% of the variation, respectively.

### Comparison of bacterial composition of two groups

As shown in **Figure 1**, there were similarities in the compositions of phyla in the two groups, but meanwhile some significant differences existed. **Supplementary Figure 3** also showed that, at phyla level, there were statistically significant differences in the abundance of seven microorganisms between groups. Firmicutes was enriched in the plateau group. However, Proteobacteria, Bacteroidetes, Candidate division TM7, Acidobacteria, Eukaryota_unclassified and Tenericutes were all significantly more abundant in the plain group (all *P* < 0.05, **Table 2**).

**Table 2:** Comparative table of the abundance of each phylum between two groups (top 10 phyla).

taxonomy	mean total	plateau group	plain group	*P* value
Firmicutes,%	58.57	75.01 ± 1.40	48.15 ± 3.66	<0.001
Proteobacteria,%	14.86	11.84 ± 0.84	22.35 ± 1.82	<0.001
Bacteroidetes,%	12.01	6.77 ± 0.77	20.45 ± 2.57	<0.001
Actinobacteria,%	4.32	4.17 ± 0.55	5.27 ± 0.68	0.184
Fusobacteria,%	1.21	0.84 ± 0.19	1.59 ± 0.72	0.399
Synergistetes,%	0.52	0.41 ± 0.07	0.71 ± 0.23	0.206
Candidate division TM7,%	0.41	0.33 ± 0.05	0.59 ± 0.11	0.044
Acidobacteria,%	0.17	0.14 ± 0.02	0.23 ± 0.03	0.013
Eukaryota_unclassified,%	0.10	0.05 ± 0.01	0.15 ± 0.03	0.005
Tenericutes,%	0.09	0.07 ± 0.01	0.13 ± 0.02	0.043

P < 0.05, the differences between groups were statistically significant.

Venn diagram can be used to depict the number of common and unique OTU or species, which can intuitively show the overlap of core microbiome between the two groups. Each group contained multiple samples, and if the number of samples of a certain OTU or species detected reached more than half (≥50%) of the total number of samples in the group, this group was considered to contain this OTU or species. We identified 114 species in each group. Among them, 107 species were uniform, occupying 88.43% of all the species detected, which suggested a steady composition of the microbiome in oral taken from both plateau and plain males. The other 14 species were not shared in two groups, and were considered to be variable microbiomes. 7 species could be found only in the plateau group, and they were *Blastococcus*, *Gemmatimonadaceae unclassified*, *Lactobacillus*, *Sphingomonas*, *Rhodobium*, *Akkermansia*, and *Bacteroides*. while there were also 7 species unique to the plain group, which were *Allisonella*, *Frankiales unclassified*, *Micrococcales unclassified*, *Hyphomonadaceae unclassified*, *Butyrivibrio*, *Spirochaetaceae unclassified*, and *Acinetobacter* (**Supplementary Figure 4**).

**Figure 3:**
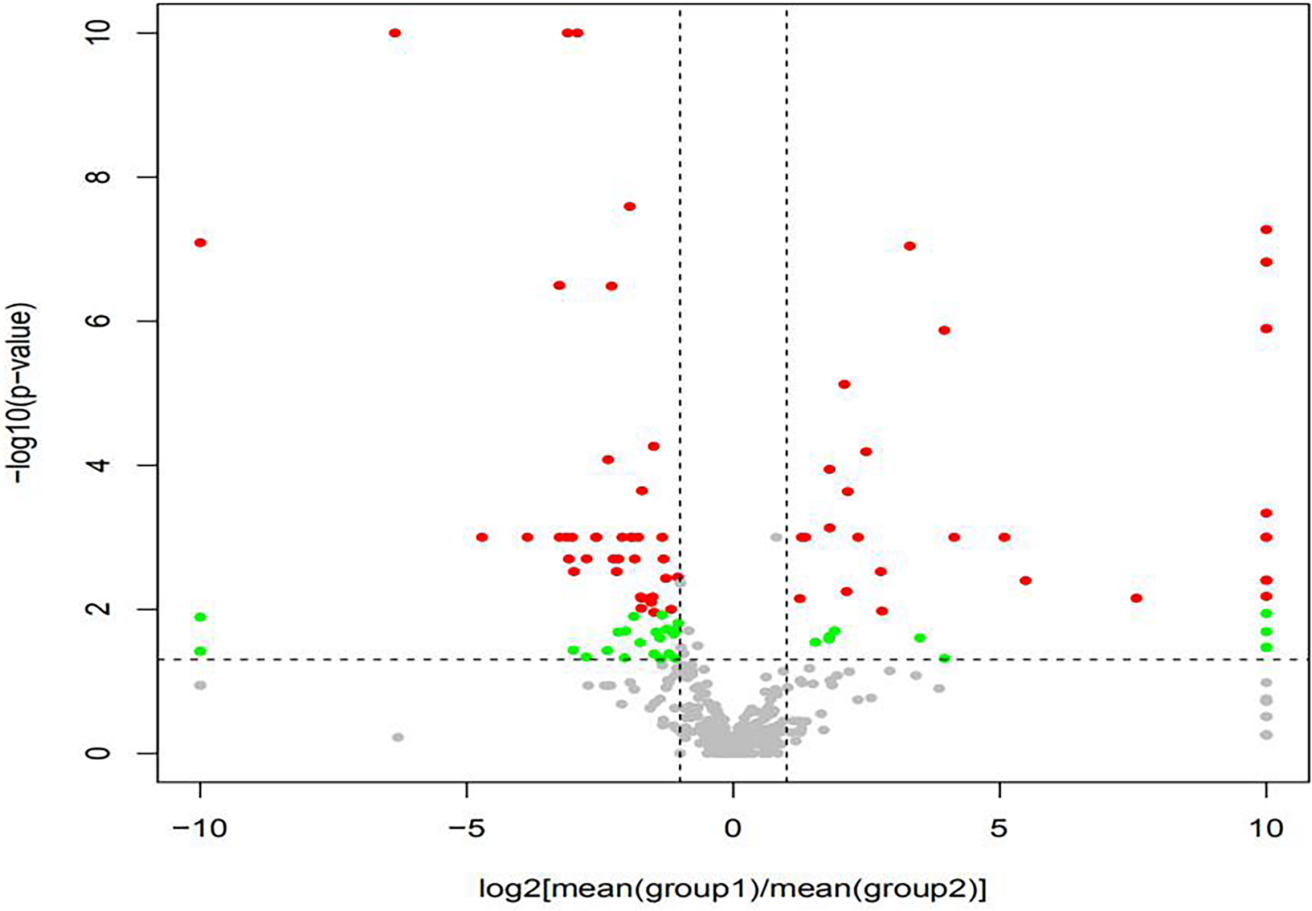
The volcano map showed the difference at genera level {Red dot: met log_2_[mean(A)/mean(B)] > 1 and *p* < 0.05 and *q* < 0.05 (advanced standard). green dot: only met log_2_[mean(A)/mean(B)] > 1 and *p* < 0.05 (primary standard). Gray dot: Taxonomy that did not meet any of the above criteria.}.

**Figure 4:**
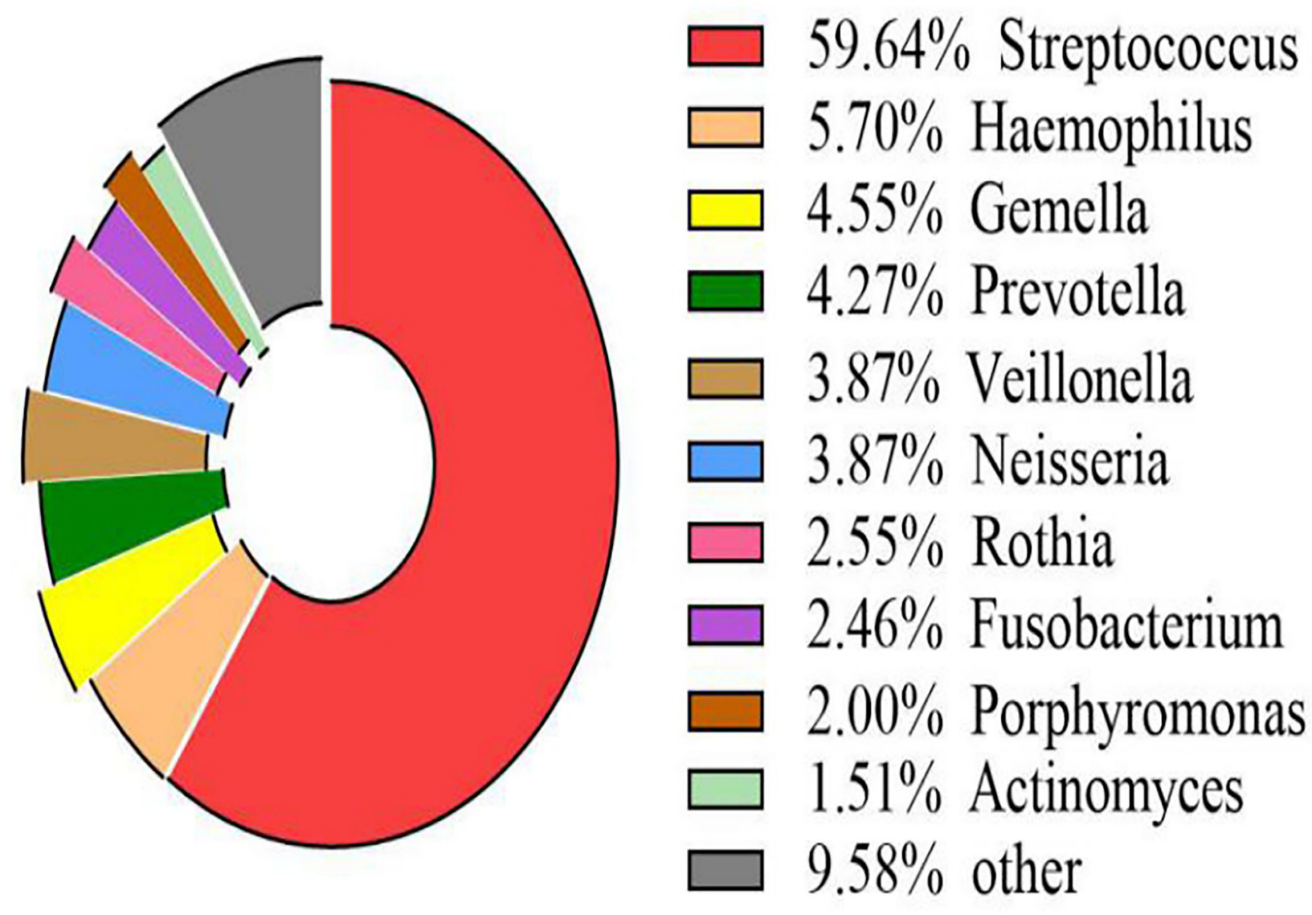
Scale map of the top 10 genera in abundance (all samples) The map displayed the proportion of several kinds of genera with richer contents directly.

**Figure 5:**
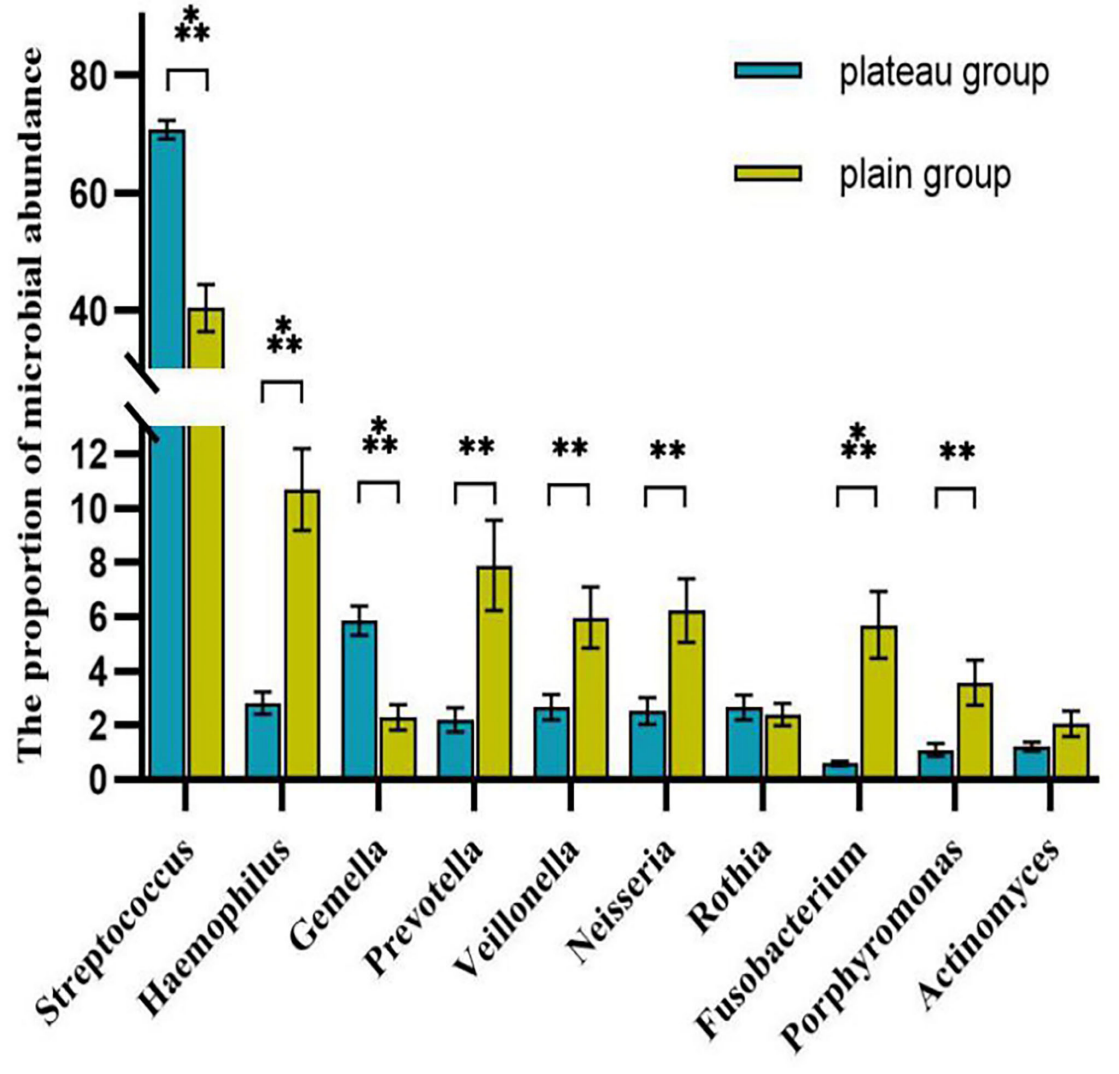
Comparison of the abundance of each genera between two groups (top 10 genera) The symbol above the columnar band indicated that the difference between groups was statistically significant. ***P < 0.001, **P < 0.01. No statistical differences were found between groups without symbols.

The volcano map showed the statistical differences in the species distribution at genera level between the two groups (**Figure 3**). We took the top 10 bacteria genera in abundance for comparative analysis, which accounted for more than 90% of the total (**Figure 4**). Streptococcus and Gemella were significantly enriched in plateau group, while Haemophilus, Prevotella, Veillonella, Neisseria, Fusobacterium and Porphyromonas all exhibited relatively higher abundance in the plain group (all *P* < 0.05, **Supplementary Table 1** and **Figure 5**).

### Function prediction

**Figure 6:**
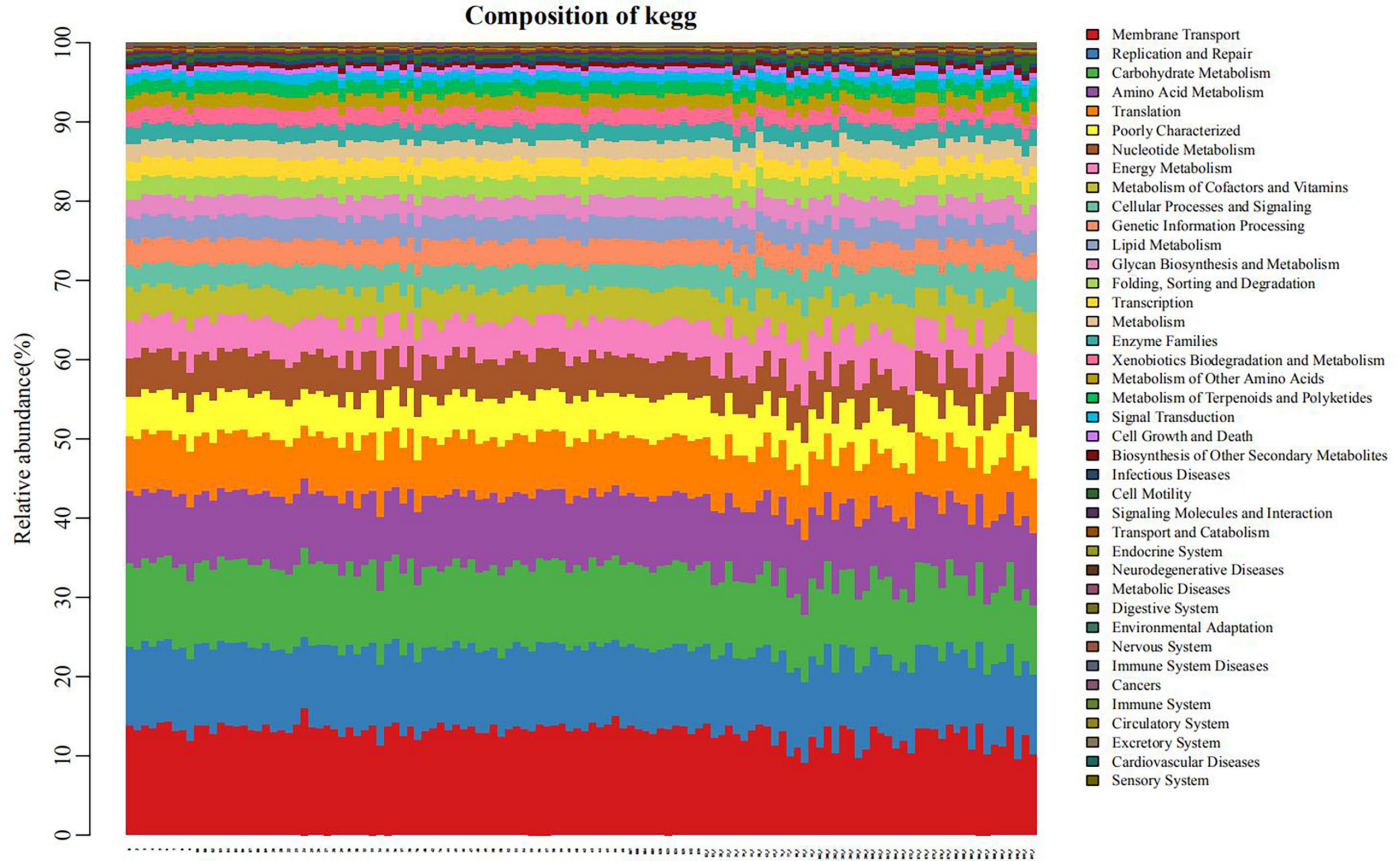
Bar diagram of the distribution of pathways and functions (the left two-thirds of the abscissa: plateau group, the right third: plain group.) Each position on the horizontal axis represented separate object. Different colors indicated different pathways or functions, and the length of each color stripe indicated the proportion of a particular pathway (function) in somebody.

The PICRUSt program was performed to help obtain information about the function profile of the microbiome in oral by 16S rRNA gene sequence. As shown in **Figure 6**, the function distribution of each sample in the plain group (the right third of the graph) was significantly different from that in the plateau group (the left two-thirds of the figure). After the correlation analyses between different bacteria and gene functions, we revealed immune system was positively correlated with the relative abundance of *Prevotella* (r=0.78, *P* < 0.05). And the relative abundance of *Streptococcus* was positively correlated with carbohydrate metabolism, cell growth and death, digestive system, infectious diseases, membrane transport, signaling molecules and interaction, xenobiotics biodegradation and metabolism, nucleotide metabolism, transcription and translation, respectively (all r > 0.7, all *P* < 0.05), negatively with the possibility of cancers (r=-0.74, *P* < 0.05). Besides, *Neisseria* relative abundance was positively associated with circulatory system and neurodegenerative diseases (both r > 0.8, both *P* < 0.05, **Supplementary Table 2**).

### Correlation analysis between oral microbiota and common blood indicators

In the plateau group, *Streptococcus* relative abundance was positively correlated with triglyceride level significantly (r=0.247, *P*=0.027). In the plain group, hemoglobin level was negatively correlated with the relative abundance of *Prevotella* (r=-0.558) and *Veillonella* (r=-0.347), both *P* < 0.05. Similarly, the number of RBC correlated negatively with *Rothia* relative abundance (r=-0.388, *P*=0.013), and the eosinophil ratio negatively with *Gemella* relative abundance (r=-0.325, *P*=0.041). In addition, the relative abundance of *Haemophilus* correlated positively with levels of aspartate transaminase (r=0.365) and mean corpuscular volume (r=0.314), both *P* < 0.05. *Fusobacterium* relative abundance and the neutrophil ratio also had a positive correlation (r=0.419, *P*=0.007). The above results were shown in **Table 3**.

**Table 3:** Correlation between bacteria abundance and blood indices in different groups.

subgroup	taxonomy	blood indices	r	*P* value
Plateau group	Streptococcus	TG	0.247	0.027
Plain group	Prevotella	Hb	-0.558	<0.001
	Rothia	the number of RBC	-0.388	0.013
	Veillonella	Hb	-0.347	0.028
	Haemophilus	AST	0.365	0.021
		MCV	0.314	0.049
	Gemella	EOSR	-0.325	0.041
	Fusobacterium	NEUR	0.419	0.007

TG, triglyceride; Hb, haemoglobin; RBC, red blood cell; AST, aspartate transaminase; MCV, mean corpuscular volume; EOSR, eosinophil ratio; NEUR, neutrophil ratio. P < 0.05: the correlation between the two variables was statistically significant.

## Discussion

Researchers are continually learning more about human microbes. Currently, microorganisms in the human body are thought to participate in physiological processes such as regulating body metabolism, promoting nutrient absorption, and adjusting immune function, and are thought to be associated with the occurrence and development of various local and systemic diseases (Dominguez-Bello et al., 2019). Li et al. (2019) found that oral microflora could colonize the intestinal tract of sterile mice through physical and chemical barriers. Evidence suggests that oral microbiota can either directly affect health or can indirectly affect health by changing the structure of intestinal microbiota (Lamont et al., 2018). Compared with intestinal flora, oral flora can be sampled more conveniently and is more amenable to direct intervention (Zhang et al., 2015). This study examined the composition and structure of oral bacterial communities in humans living at different altitudes and ambient oxygen levels, allowing us to better understand human adaptation or non-adaptation to living on the plateau versus the plain. The relationship between oral microbiota and common blood indices was also explored. Because the oral microbiome changes as people age (Jiang et al., 2018), we selected research subjects who were all young men of approximately the same age.

In addition to age, other potentially confounding factors such as ethnicity and teeth-brushing habits were similar between the two groups, which allowed us to eliminate extraneous variables in our investigation of altitude as a factor. Our results showed that the oral microbiota in the plain group was both more diverse and more evenly distributed among species than the oral microbiota in the plateau group. These findings were consistent with the results of a previous study on ocular microbiota (Li et al., 2021). Other studies have shown that more diverse bacterial communities may correspond to healthier ecosystems (Xiao et al., 2016). Because the higher species diversity buffered the variability of ecosystem function, biodiversity is essential for promoting the sustainability and productivity of many ecosystems (Bannar-Martin et al., 2018). Principal coordinates analysis (PCoA) on unweighted UniFrac distances revealed that different species within the bacterial community characterize the two altitudes. However, no more than 10% variation between the two groups was displayed in any coordinate direction, indicating that the plateau and plain groups had similar, although not identical, oral bacterial community structures. A Venn diagram showed the same species accounted for about 85% of the total number of species detected, also suggesting that the species composition of the oral microbiome was similar between the two groups.

The dominant bacteria in any one part of the body generally remain relatively stable. The species that occur more frequently, in greater abundance, and more consistently over time are referred to as the “core microbiome” of that particular part of the body (Hu et al., 2013). Firmicutes was the most abundant phylum in both the plain group and the plateau group, indicating the bacterial community structure is relatively consistent in the oral cavity, but the altitude factor may affect the abundance of Firmicutes (75% in the plateau group vs 48% in the plain group). This finding is similar to that of a previous study, which found that cold acclimation resulted in significant changes in microbiota composition (Bo et al., 2019). However, the abundance of Firmicutes in the plain group was lower than that in the plateau group, which is consistent with the higher richness and more even species distribution of the oral microbiota in the plain group. Some scholars believe that Firmicutes can encode enzymes related to energy metabolism, and produce a variety of digestive enzymes which decompose various substances (Turnbaugh et al., 2006; Li and Zhao, 2015). So it is convenient to save the body energy consumption and promote metabolism, suggesting that higher energy harvest and expenditure existed in populations at higher altitudes.

The other four phyla among the top five were Proteobacteria, Bacteroidetes, Actinobacteria, and Fusobacteria. Among them, the proportion of Proteobacteria and Bacteroidetes in the plain group was higher than that in the plateau group, while the proportion of Actinobacteria and Fusobacteria in the two groups showed no statistical difference. At the genus level, *Streptococcus* spp. was most abundant, accounting for 70.76% and 40.44% of the total abundance in the plateau group and the plain group, respectively. In order of abundance, the other dominant genera were, *Haemophilus*, *Gemella*, *Prevotella*, *Veillonella*, *Neisseria*, *Rothia*, *Fusobacterium*, *Porphyromonas*, and *Actinomyces*. Among these nine genera, the relative abundance of *Gemell* spp. was higher in the plateau group than in the plain group, whereas the abundances of the other eight genera in the plateau group were either lower than or not statistically different from those in the plain group. The microbiome differences found in this study were broadly consistent with those in other studies (Senapati et al., 2019; AlShahrani et al., 2020). However, unlike our study, Das B et al. (Das et al., 2018) found that the abundance of *Prevotella* spp. was higher in the plateau population. This difference may be related to race and/or oral health. Our findings regarding variations in abundance among different species should be further verified by conducting a follow-up study using a larger sample size.

The reasons for the difference in the abundance of bacteria between the two groups were attempted to analyze. Firstly, the microbial flora, richer in the high altitude environment, can convert materials into short-chain fatty acids (SCFA) through microbial fermentation. SCFA is an important energy source for epithelial cells, providing about 10% of human energy (Tremaroli and Bäckhed, 2012). Increased abundance of specific flora in the plateau appears to compensate for reduced energy intake and increased energy expenditure. Secondly, low temperature reduces the activity of microorganisms and enzymes at high altitude, which may limit the abundance of some bacteria (Wang et al., 2015). And the increase in the abundance of Bacteroidetes, Candidate division TM7 and other bacteria may be the result of their increased nutrient utilization with the warming of temperature (Schostag et al., 2019). Thirdly, hypoxia can induce and activate oxidative stress and inflammatory responses in the host, resulting in changes in the composition and decrease diversity of the microbiome (Lucking et al., 2018). Moreover, the decrease in Bacteroidetes in the plateau group may be related to the lower intake of fruit (Wu et al., 2020), that is, the objective restriction of food storage and production conditions resulted in different dietary habits in the two regions. Therefore, we hypothesized that dietary structure, maximal oxygen uptake, cold and hypoxia contributed to the differences in microbial abundance at different altitudes.

The abundance of bacteria only present in one of the two groups was very low, so their presence may have no obvious effect on the human body. The possible reasons that some microbiome have only been found to exist in certain altitude include: Allisonella belongs to the nutritional fastidious group (Seshadri et al., 2018). And it tends to be found in plain areas where material conditions are abundant; Frankiales unclassified and Acinetobacter were more abundant in the plastic (Wright et al., 2021), moreover, people on the plains are more likely to have access to household or industrial plastics; Hyphomonadaceae unclassified is an aerobic, heterotrophic bacteria genus, which is generally isolated from surface drainage (Podell et al., 2020); Aciditerrimonas, Blastococcus and Sphingomonas were found in the desert (Wang et al., 2017). In most habitats, environment had a strong influence on bacterial community composition, suggesting that selection may play an important role in shaping the biogeographic pattern of microorganisms in the cold plateau regions; The survival of Lactobacillus and biological fermentation may correspond to the hypoxic environment of the plateau (Call et al., 2018). In conclusion, we suspect that the occurrence of these bacteria only in one group may be related to the dry, cold and oxygen-deficient environment and residents’ living habits.

These results suggest that the effect of altitude on the oral microbiome is mainly due to changes in the community structure of the hosts’ existing internal microorganisms, rather than to new microbial colonization. The PICRUSt program was used for function prediction. All groups showed similar microbial functional characteristics, which may be influenced by the widely distributed core microbiome. By comparing the relative abundance of functional categories, we found that membrane transport, replication and repair, carbohydrate metabolism, and amino acid metabolism were abundant, indicating that microbial metabolism was vigorous in the oral microbiome. At high altitudes, low oxygen levels and high ultraviolet exposure can lead to DNA and protein damage (Foll et al., 2014), and enrichment of genes related to translation, amino acid metabolism and other functions mentioned above may help reduce biomolecular damage. *Streptococcus* spp., with the highest abundance in the plateau group, was closely related to a variety of metabolic pathways. This finding is consistent with the commonly held opinion of many researchers that the microbiota in people living at higher altitudes is more energy-efficient than the microbiota in their counterparts living at lower altitudes (Li et al., 2016; Li et al., 2021). This suggests the oral microbiome may be one potential mediator of plateau hypoxia-mediated metabolic imbalance (Pasiakos et al., 2021).

Because the core microbiome is relatively stable in a specific segment of the population, it raises the possibility of using oral microbiota as biomarkers for changes in common blood parameters. In this study, we found that the abundance of *Streptococcus* spp. was positively correlated with triglyceride levels in the plateau group. Given that triglycerides are closely associated with cardiovascular disease (CVD) (Crea, 2021), this result indicates that changes in the abundance of this bacterium are not only related to altitude adaptation but could also be related to CVD induced by altitude (Ha et al., 2021). Similar studies have suggested that the microbiome may play an important role in changing lipid levels (Fu et al., 2015). In a study investigating the effects of the Mediterranean diet on microbiota, Wang et al. (2021) found that high *Prevotella* spp. abundance was often accompanied by high triglycerides. It remains uncertain whether this change in blood lipid levels is merely a derivative adaptation to a harsh hypoxic environment or is directly caused by specific microbial changes. In general, the effects of bacterial flora on blood lipids may be related to dietary fermentation and the resulting metabolites. Nevertheless, in the plain group, we found that *Prevotella* spp., *Rothia* spp., and *Veillonella* spp. were negatively correlated with erythrocyte-related indicators, which could coincide with the decrease in activity of erythrocyte Na^+^/K^+^-ATPase (Yin et al., 2021). We found a positive correlation between the abundance of *Haemophilus* spp. and aspartate transaminase (AST) levels, while Sun et al. (2021) reported a negative correlation between the abundance of Saccharomyces cerevisiae in the Chinese urban population and levels of AST related to liver function. The specific functions of these bacterial species and their relationships to human health need further study.

We acknowledge the limitations in this preliminary study. First, our method of collecting oral samples did not distinguish among the different areas of the oral ecological region, so it was impossible to study specific oral sites and examine the unique flora characteristics of subgingival plaque, saliva, or other parts of the subject’s mouth. Second, participants’ periodontal and dental health assessment was not complete, which may have affected the analysis of bacterial flora structure. Third, the study only included young men, so the conclusions cannot be applied to all populations. Finally, this study cannot exclude the influence of dietary structure and individual differences in saliva flow on oral microbiota.

## Conclusion

In summary, this study comprehensively analyzed the microbiota of different ecological niches, the influence of altitude on oral microbiota, and the possible correlation between specific bacteria and blood indicators. Our results showed that the diversity of oral microbiota was higher in the plain (low altitude) group than in the plateau (high altitude) group, and the difference between the two groups was mainly reflected in the relative proportion of predominant bacteria in each region. This study provides a point of reference for studying the biological mechanism of acclimatization or maladaptation to high altitude.

## Data availability statement

All relevant data is contained within the article: The original contributions presented in the study are included in the article/supplementary material, further inquiries can be directed to the corresponding author/s.

## Ethics statement

The studies involving human participants were reviewed and approved by the ethics committee of Chinese PLA General Hospital (S2020-363-01). The patients/participants provided their written informed consent to participate in this study.

## Author contributions

LL and LF contributed to the research design, data interpretation, and critically revised the manuscript. KC and YG contributed to the research design and data acquisition. HW, XL, and RW collected the data and the samples. LZ contributed to data interpretation and wrote the manuscript. BH contributed to design and make figures. All authors gave final approval and agreed to be accountable for all aspects of this work.

## Funding

This study was supported by Youth Program for Military Medicine of Chinese PLA General Hospital (QNC19054), Military Health Care Project (19BJZ34), and Military Equipment Construction Application Research Project (LB20211A010013). The funders had no direct role in the design, data collection, analysis, interpretation, or writing of the manuscript.

## Acknowledgments

We acknowledge Beijing Qinglian Biotech Co., Ltd. for their kind help with Illumina MiSeq sequencing and guidance and collaboration on data analysis.

## Conflict of interest

The authors declare that the research was conducted in the absence of any commercial or financial relationships that could be construed as a potential conflict of interest.

## Publisher’s note

All claims expressed in this article are solely those of the authors and do not necessarily represent those of their affiliated organizations, or those of the publisher, the editors and the reviewers. Any product that may be evaluated in this article, or claim that may be made by its manufacturer, is not guaranteed or endorsed by the publisher.
