# Supplementary material for: Characteristics of oral microbiota in plateau and plain youth‐positive correlations between blood lipid level, metabolism and specific microflora in the plateau group
Source: Front Cell Infect Microbiol. 2022 Aug 10;12:952579. doi: 10.3389/fcimb.2022.952579 (PMC9400057; doi:10.3389/fcimb.2022.952579)
Supplement: Supplementary file 1 [file DataSheet_1.docx]

Supplementary Tab.1 Comparative table of the abundance of each genera between two groups (top 10 genera)

| taxonomy | mean total | plateau group | plain group | *P* value |
| --- | --- | --- | --- | --- |
| Streptococcus,% | 59.64 | 70.76±1.61 | 40.44±4.04 | <0.001 |
| Haemophilus,% | 5.70 | 2.81±0.41 | 10.68±1.51 | <0.001 |
| Gemella,% | 4.55 | 5.85±0.54 | 2.29±0.47 | <0.001 |
| Prevotella,% | 4.27 | 2.19±0.44 | 7.88±1.66 | 0.002 |
| Veillonella,% | 3.87 | 2.65±0.47 | 5.96±1.13 | 0.010 |
| Neisseria,% | 3.87 | 2.51±0.49 | 6.22±1.17 | 0.002 |
| Rothia,% | 2.55 | 2.65±0.45 | 2.39±0.41 | 0.676 |
| Fusobacterium,% | 2.46 | 0.59±0.08 | 5.69±1.23 | <0.001 |
| Porphyromonas,% | 2.00 | 1.09±0.23 | 3.57±0.83 | 0.007 |
| Actinomyces,% | 1.51 | 1.20±0.16 | 2.05±0.46 | 0.079 |

Supplementary Tab.2 Predicting the gene function of the corresponding bacteria

| taxonomy | pathway and function | r | *P* value |
| --- | --- | --- | --- |
| Prevotella | immune system | 0.78 | 1.61E-25 |
| Streptococcus | cancers | -0.74 | 3.26E-22 |
|  | carbohydrate metabolism | 0.76 | 2.01E-23 |
|  | cell growth and death | 0.71 | 8.62E-20 |
|  | digestive system | 0.82 | 1.55E-30 |
|  | infectious diseases | 0.92 | 2.37E-51 |
|  | Membrane transport | 0.77 | 4.44E-25 |
|  | nucleotide metabolism | 0.74 | 1.81E-22 |
|  | signaling molecules and interaction | 0.94 | 3.32E-56 |
|  | transcription | 0.71 | 6.32E-20 |
|  | translation | 0.73 | 2.01E-21 |
|  | xenobiotics biodegradation and metabolism | 0.78 | 7.52E-26 |
| Neisseria | circulatory system | 0.97 | 3.60E-79 |
|  | neurodegenerative diseases | 0.84 | 5.63E-33 |


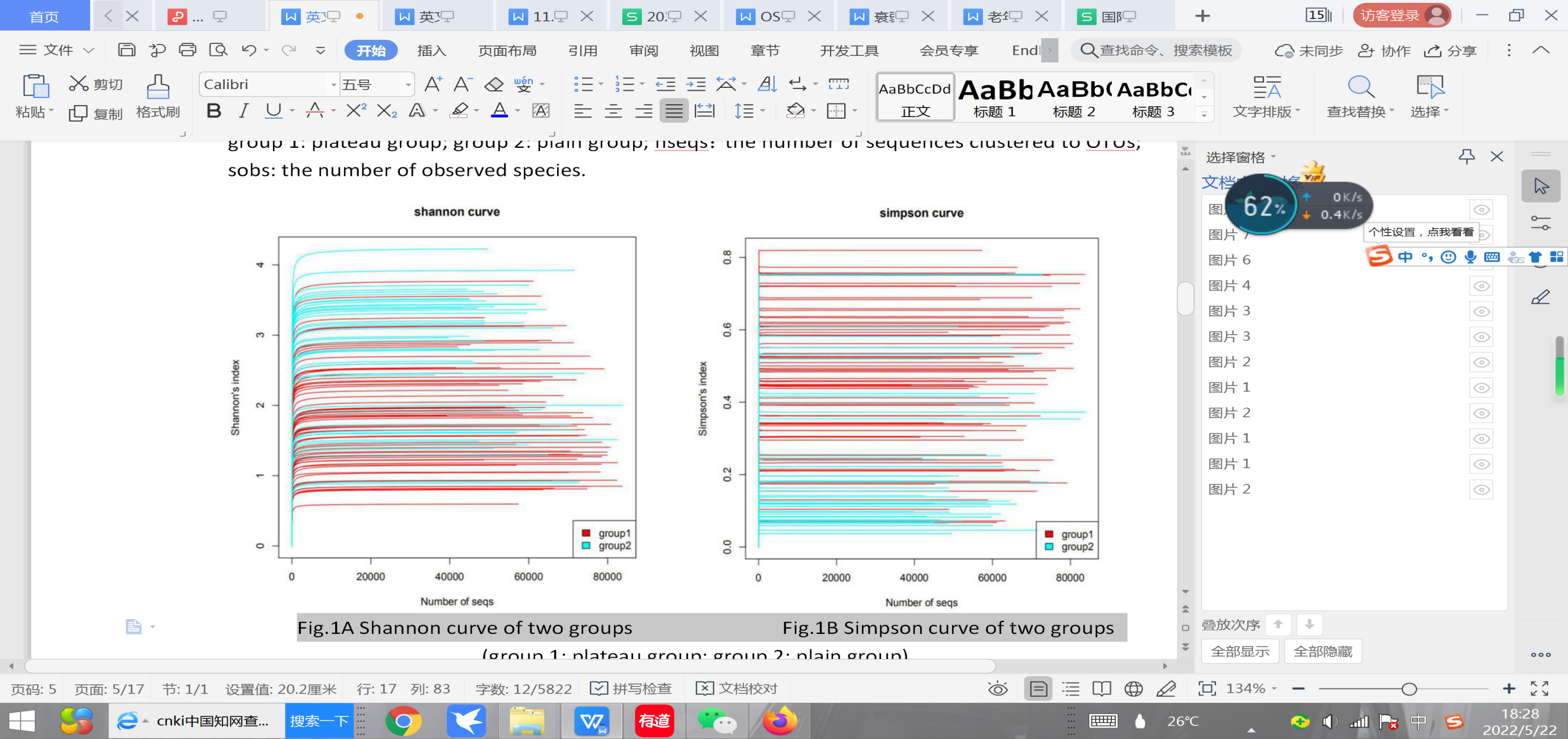


Supplementary Fig.1A (Left) Shannon curve of two groups, 1B (Right) Simpson curve of two groups (group 1: plateau group; group 2: plain group)


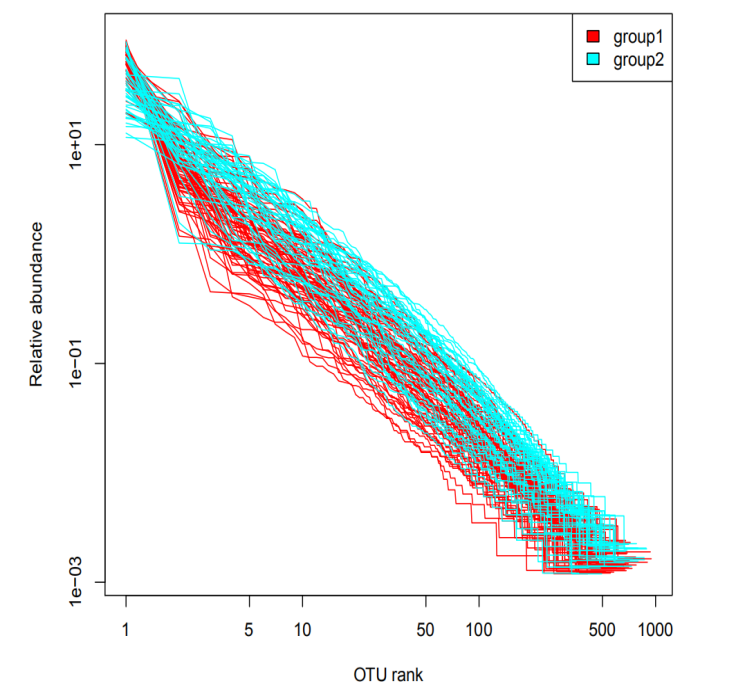


Supplementary Fig.2 Rank Abundance curve (group 1: plateau group; group 2: plain group)


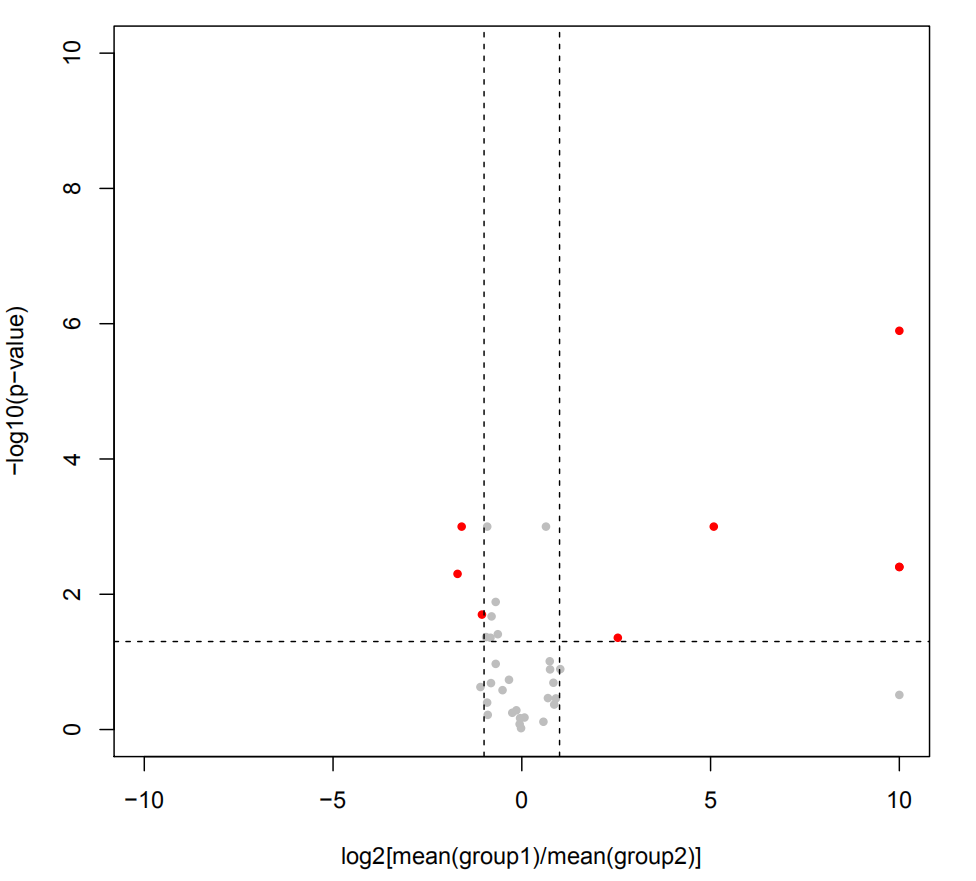


Supplementary Fig.3 The volcano map showed the difference at phyla level


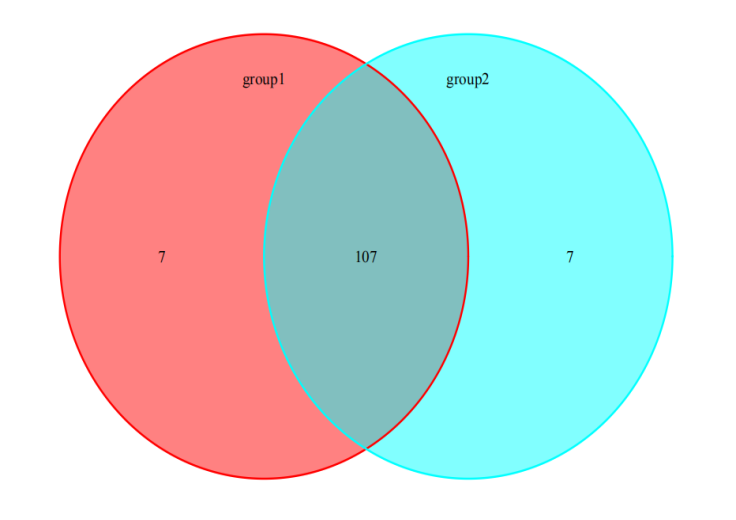


Supplementary Fig.4 Venn diagram at the species level (group1: plateau group; group2: plain group)
